# Effect of end-stage kidney disease on the return of spontaneous circulation in Taiwanese adults with out-of-hospital cardiac arrest

**DOI:** 10.1038/s41598-023-35024-8

**Published:** 2023-05-16

**Authors:** Ming-Shun Hsieh, Amrita Chattopadhyay, Tzu-Pin Lu, Shu-Hui Liao, Chia-Ming Chang, Yi-Chen Lee, Wei-En Lo, Jia-Jun Wu, Vivian Chia-Rong Hsieh, Sung-Yuan Hu, Chorng-Kuang How

**Affiliations:** 1 Department of Emergency Medicine, Taipei Veterans General Hospital, Taoyuan Branch, Taoyuan, Taiwan; 2 Department of Emergency Medicine, Taipei Veterans General Hospital, Taipei, Taiwan; 3 School of Medicine, National Yang Ming Chiao Tung University, Taipei, Taiwan; 4 Department of Emergency Medicine, Taichung Veterans General Hospital, Taichung, Taiwan; 5 Bioinformatics and Biostatistics Core, Center of Genomics and Precision Medicine, National Taiwan University, Taipei, Taiwan; 6 Department of Public Health, National Taiwan University, Taipei, Taiwan; 7 Department of Pathology and Laboratory, Taipei Veterans General Hospital, Taoyuan Branch, Taoyuan, Taiwan; 8 Institute of Occupational Medicine and Industrial Hygiene, National Taiwan University, College of Public Health, Taipei, Taiwan; 9 Department of Critical Care Medicine, Taipei Veterans General Hospital, Taoyuan Branch, Taoyuan, Taiwan; 10 Department of Health Services Administration, China Medical University, Taichung, Taiwan; 11 School of Medicine, Chung Shan Medical University, Taichung, Taiwan; 12 Institute of Medicine, Chung Shan Medical University, Taichung, Taiwan; 13 Department of Post‐Baccalaureate Medicine, College of Medicine, National Chung Hsing University, Taichung, Taiwan

**Keywords:** Kidney diseases, Interventional cardiology

## Abstract

Rescuing patients with out-of-hospital cardiac arrest (OHCA), especially those with end-stage kidney disease (ESKD), is challenging. This study hypothesizes that OHCA patients with ESKD undergoing maintenance hemodialysis have (1) higher rates of return of spontaneous circulation (ROSC) during cardio-pulmonary resuscitation (CPR) and (2) lower rates of hyperkalemia and less severe acidosis than those without ESKD. OHCA patients who received CPR between 2011 and 2020 were dichotomized into ESKD and non-ESKD groups. The association of ESKD with “any” and “sustained” ROSC were examined using logistic regression analysis. Furthermore, the effect of ESKD on hospital outcomes for OHCA patients who survived to admission was evaluated using Kaplan–Meier analysis. ESKD patients without “any” ROSC displayed lower potassium and higher pH levels than non-ESKD patients. ESKD was positively associated with “any” ROSC (adjusted-OR: 4.82, 95% CI 2.70–5.16, *P* < 0.01) and “sustained” ROSC (adjusted-OR: 9.45, 95% CI 3.83–24.13, *P* < 0.01). Kaplan–Meier analysis demonstrated ESKD patients had a non-inferior hospital survival than non-ESKD patients. OHCA patients with ESKD had lower serum potassium level and less severe acidosis compared to the general population in Taiwan; therefore, should not be treated under the stereotypical assumption that hyperkalemia and acidosis always occur.

## Introduction

Out-of-hospital cardiac arrest (OHCA) is a major public health concern, with an average incidence of 55 per 100,000 person-years among adults worldwide^1^. From a meta-analysis conducted by Yan et al. in 2020, the pooled incidence of return of spontaneous circulation (ROSC) was found to be 29.7% (95% confidence interval (CI) 27.6–31.7%), the rate of survival to hospital admission was 22.0% (95% CI 20.7–23.4%), and the rate of survival to discharge was 8.8% (95% CI 8.2–9.4%). In addition, the pooled 1-month and 1-year survival rates were 10.7% (95% CI 9.1–13.3%) and 7.7% (95% CI 5.8–9.5%), respectively^2^. However, survived to discharge is not synonymous with entire recovery, and only 3–7% of OHCA survivors regained their pre-cardiac arrest function^3,4^. Even if ROSC is achieved on time, certain psychological effects, such as anxiety, depression, and post-traumatic stress disorder may persist^5^.

In Taiwan, the prevalence of chronic kidney disease (CKD) was high, accounting for approximately 11.9% of the total population in 2008^6^. According to the US Renal Data System (USRDS) 2021 report, Taiwan was one of the three countries with the highest rate of increase in the number of patients with end-stage kidney disease (ESKD) who required long-term dialysis^7^. And, it was estimated that about 91.4% of ESKD patients received hemodialysis (HD), and 8.6% received PD^8^. From 2009 to 2013, the 1, 3, and 5-year cumulative survival rates of ESKD patients undergoing maintenance hemodialysis were 89.4%, 69.6%, and 54.2%, respectively^9^.

The association between ESKD and the OHCA occurrence had been extensively reported^10–12^. But the effect of ESKD on the OHCA patients, including ROSC rate and hospital outcome was little discussed. Furthermore, there was no study including laboratory data currently (such as potassium level and PH value) in the ESKD and non-ESKD OHCA patients. In general, the ESKD patients in Taiwan received HD in the manner of three sections per week. In clinical practice, we frequently observed that the OHCA patients with ESKD were more likely to attain “any” and “sustained” ROSC during Cardio-Pulmonary Resuscitation (CPR) compared to the general population. However, we found no evidence in the literature to support it.

Sodium bicarbonate (SB) administration, as a part of the treatment for severe metabolic acidosis in OHCA, was essential because it allowed the normalization of extracellular and intracellular pH as a pathophysiological endpoint of resuscitation. However, the safety and effectiveness of SB administration during OHCA had been debated for decades. The latest Advanced Cardiac Life Support (ACLS) guidelines in 2020 did not recommend the routine use of sodium bicarbonate for patients experiencing cardiac arrest except for hyperkalemia or tricyclic anti-depressant overdose^13^. The latest meta-analysis study also demonstrated that SB is not associated with increased ROSC and survival to discharge rate; furthermore, SB was found to be associated with lower rates of sustained ROSC and good neurological outcomes. However, Niederberger et al. in 2023 demonstrated reverse findings of improved survival in asystolic and pulseless electrical activity (PEA) OHCA patients by the administration of SB^14^.

This study aims to examine whether ESKD has a positive association with ROSC. According to our observations in clinical practice, we hypothesized that prolonging CPR in patients with ESKD during OHCA would result in higher rates of ROSC; thus in this study, we questioned the conventional understanding that hyperkalemia and acidosis act as barriers. We used long-term (2010–2020) hospital-based ED data to examine the odds of ROSC among patients with and without ESKD after OHCA and assessed the associative nature of serum potassium and pH levels in the ESKD group after OHCA.

## Methods

### Study population

**Figure 1:**
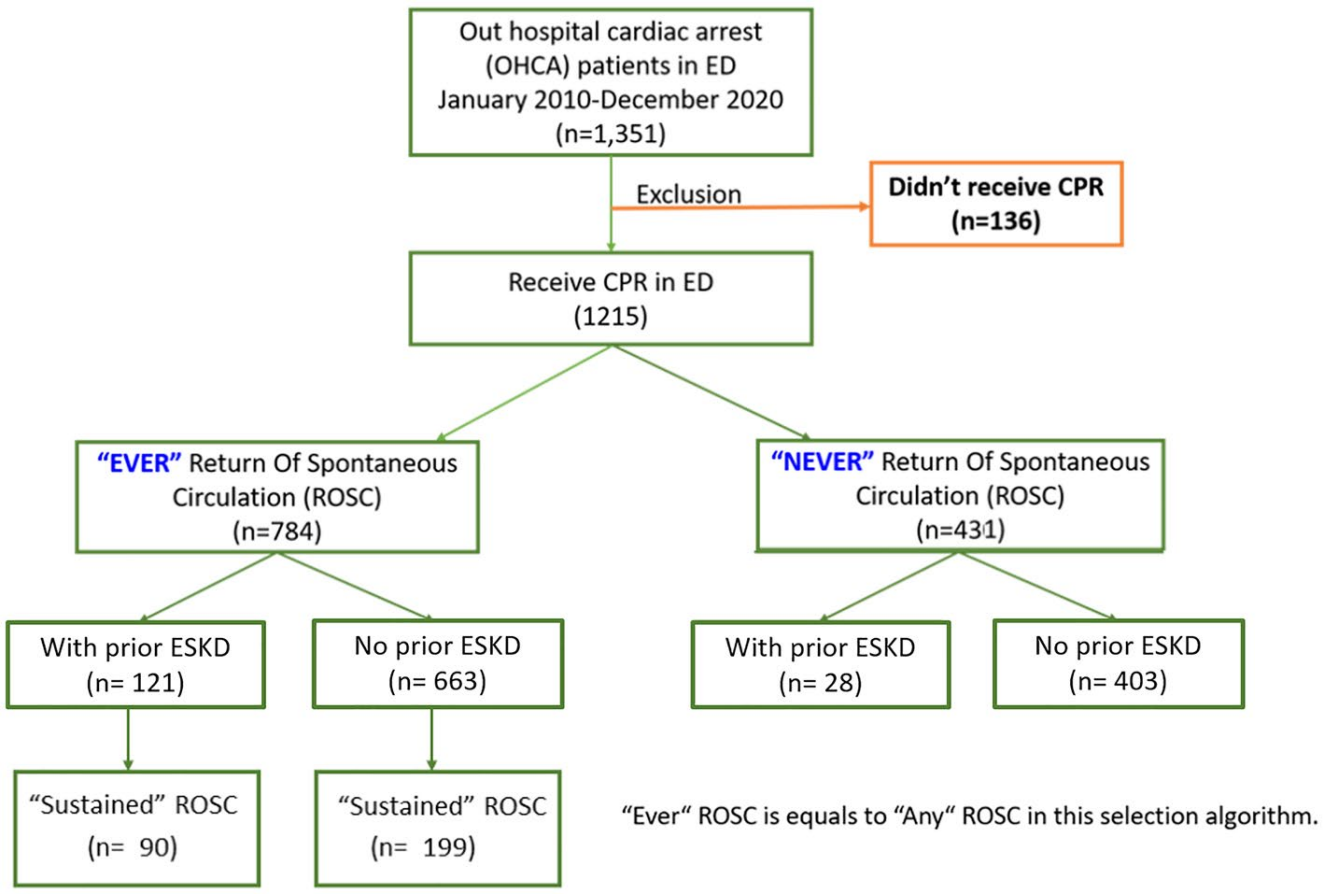
Participant exclusion and inclusion criteria.

This study was conducted in Taichung Veterans General Hospital, a medical center in central Taiwan. Using the hospital’s medical database dated from January 2010 to December 2020, we retrospectively identified Taiwanese adults with OHCA. In the standard template of Utstein registry, OHCA etiology was classified into seven categories: medical, trauma, overdose, drowning, electrocution, asphyxia, and not recorded^15^. In our study, we only enrolled patients with OHCA belonging to the “medical” category. Out of 1351 patients with OHCA, 1215 who received CPR in the ED were included in the analysis. All 1215 patients suffered from OHCA and reached the hospital with ongoing CPR. The selection algorithm is demonstrated in Fig. 1.

Patients with OHCA who were transferred from other hospitals were excluded from this study. This was to eliminate the possible bias due to these patients receiving several medications, which might interfere with the interpretation of the study observations. Additionally, patients with OHCA, transferred directly from the HD clinics were excluded. Only epinephrine injections are allowed by the emergency medical service (EMS) system in Taiwan, while usage of sodium bicarbonate is not. This study was approved by the institutional review board of Taichung Veterans General Hospital (IRB No. CE21215A). As the patient identification were scrambled to ensure privacy before the release of the data, this study was exempted from informed consents from participants.

### Study variables

Information such as demographic, baseline comorbidities, laboratory results, emergency managements, clinical courses, and hospital outcomes for each of the study subjects were collected for this study. All laboratory data of patients with OHCA were obtained before starting resuscitation at the ED. We present continuous variables as median ± interquartile range (IQR) and categorical variables as the number and percentage of the total participants. Patients with OHCA who received CPR were further divided into “non-ESKD” group (patients who never received HD) and the “ESKD” group. According to the KDIGO (Kidney Disease: Improving Global Out-comes) guidelines, the ESKD patients in this study were those who suffered from kidney failure and received maintenance HD for at least 3 months^16^. Taiwan's healthcare system provides comprehensive coverage for ESKD management and each ESKD patient undergoing maintenance hemodialysis typically receives three dialysis sessions per week. Such high frequency of dialysis keeps the acidosis under control and adjusts the electrolyte levels. The subset of patients receiving daily maintenance HD or those in precarious condition were excluded from this study. Those with ESKD undergoing peritoneal dialysis (PD) were further excluded to avoid unknown confounding effects. “Any ROSC” (or “ever ROSC”) were used to define the outcome where patients attained ROSC at least once during the CPR performance irrespective of the final hospital outcome. Patients were defined to have “sustained ROSC” (or “survived event”) if they survived to admission in the intensive care unit (ICU) irrespective of the frequency of cardiac re-arrest and duration of the CPR.

The binary status (yes/no) of baseline comorbidities such as hypertension, diabetes mellitus, hyperlipidemia, chronic obstructive pulmonary disease, chronic liver disease, CKD, peripheral arterial occlusive disease, stroke, ischemic heart disease, and cancer were determined in each participant. Blood marker levels such as white blood cells, hemoglobin, platelets, creatinine, sodium, potassium, troponin I, C-reactive protein, pH level, and lactate were also recorded. Furthermore, information on emergency managements, such as external defibrillation, and administration of epinephrine, amiodarone, lidocaine or sodium bicarbonate, for patients with OHCA were also included.

### Statistical analysis

All baseline characteristics, laboratory data, and medical treatments were compared between the patients with and without ESKD in the OHCA group, using Chi-square test and Wilcoxon rank-sum test, for categorical and continuous variables, respectively^17^. Univariate and multivariate adjusted logistic regression analyses were then conducted for all selected clinically important variables, with ROSC occurrence (“any” and “sustained” ROSC) as the primary outcomes. Finally, Kaplan–Meier survival analysis with a two-tailed log-rank test was conducted to evaluate the ED and hospitalization course (length of hospital stay of 28-day) in the ESKD and non-ESKD patients. Subgroup analysis which divided the OHCA patients into non-CKD, CKD, and ESKD was also performed. *P* value < 0.05 was considered statistically significant. All statistical data were analyzed using R software, where Kaplan–Meier analysis was conducted using the survival package.

### Propensity score matching

Propensity score matching (PSM) is a statistical strategy for dealing with data in observational research. Biases and confounding variables are common in observational research for a variety of reasons and PSM is employed to lessen the impact of the biases due to confounders making the comparisons between the experimental and control groups more acceptable^18–20^. In this study, PSM was performed to match and select the ESKD and non-ESKD patients in a 1:4 ratio by using “nearest neighbor matching” (greedy matching).

### Ethics approval and consent to participate

The research was carried out in accordance with the STROBE reporting guidelines for observational studies, under the EQUATOR network reporting guidelines. This work has been approved by the by the institutional review board of Taichung Veterans General Hospital, and all patients gave their informed consent (IRB # CE21215A).

## Results

### Demographic, clinical characteristics, and medical managements in the ESKD and non-ESKD groups

A total of 1251 patients with OHCA, receiving CPR treatment in the ED between January 1, 2010, and December 31, 2020, were included for analyses in this study. Out of 1251, 784 demonstrated any ROSC (or ever ROSC) and 431 did not. The rate of any ROSC was 64.52% (784/1215) out of which 121 had ESKD undergoing maintenance HD, and 663 did not. Any ROSC was attained by 81.21% (121/149) of patients in the ESKD group and 49.07% (663/1066) of the non-ESKD group. The rate of sustained ROSC later decreased to 23.78% (289/1215). Sustained ROSC was achieved by 74.38% (90/149) of the ESKD group and 30.01% (199/1066) of the non-ESKD group.

Both ESKD and non-ESKD groups had more males (67.76% and 67.57%, respectively) than females. The median (IQR) age of those with any ROSC was lower, 66.69 (17.46) years, in the ESKD group than in the non-ESKD group, 72.72 (29.75) years) (*P* < 0.01). Table 1 further demonstrated the comparison results of the baseline comorbidities, blood markers, and emergency treatment variables between the ESKD and non-ESKD groups who had OHCA with and without any ROSC. The ESKD group with any ROSC had a higher Charlson comorbidity index (CCI) Score compared to the non-ESKD group with any ROSC [CCI score 5 (IQR 5) vs. 2 (IQR 6), *P* < 0.01].

**Table 1:** Descriptive statistics of all out-of-hospital cardiac arrest (OHCA) patients with and without end-stage kidney disease (ESKD).

Variables	Out-of-hospital cardiac arrest (OHCA) patients with resuscitation (n = 1215)					
	Any ROSC = Yes (n = 784)			Any ROSC = No (n = 431)		
	ESKD (n = 121)	Non-ESKD (n = 663)	*P* value	ESKD (n = 28)	Non-ESKD (n = 403)	*P* value
Sex			1			0.47
Male	82 (67.77)	448 (67.57)		17 (60.71)	279 (69.23)	
Female	39 (32.23)	215 (32.43)		11 (39.29)	124 (30.77)	
Age (years)			< 0.01*			0.46
20–29	2 (1.75)	32 (5.06)		1 (3.57)	10 (2.53)	
30–39	3 (2.63)	24 (3.80)		2 (7.14)	15 (3.80)	
40–49	11 (9.65)	47 (7.44)		1 (3.57)	31 (7.85)	
50–59	21 (18.42)	78 (12.34)		6 (21.43)	49 (12.41)	
60–69	31 (27.19)	93 (14.72)		4 (14.29)	56 (14.18)	
70–85	36 (31.58)	205 (32.44)		11 (39.29)	149 (37.72)	
≥ 85	10 (8.77)	153 (24.21)		3 (10.71)	85 (21.52)	
Median (IQR)	66.69 (17.46)	72.72 (29.75)	< 0.01*	69.67 (19.74)	74.13 (26.48)	0.20
Baseline comorbidities						
HTN	46 (38.02)	99 (14.93)	< 0.01*	15 (53.57)	67 (16.63)	< 0.01*
DM	60 (49.59)	172 (25.94)	< 0.01*	16 (57.14)	107 (26.55)	< 0.01*
Hyperlipidemia	30 (24.79)	60 (9.05)	< 0.01*	0 (0)	1 (0.25)	1.00
COPD	28 (23.14)	173 (26.09)	0.57	11 (39.29)	95 (23.57)	0.1
CLD	17 (14.05)	50 (7.54)	0.03*	4 (14.29)	21 (5.21)	0.07
CKD	–	166 (25.04)		–	104 (25.81)	
PAOD	10 (8.26)	22 (3.32)	0.02*	6 (21.43)	21 (5.21)	< 0.01*
Stroke	45 (37.19)	181 (27.30)	0.04*	10 (35.71)	120 (29.78)	0.65
IHD	31 (25.62)	71 (10.71)	< 0.01*	9 (32.14)	45 (11.17)	< 0.01*
Cancer	30 (24.79)	209 (31.52)	0.17	7 (25)	88 (21.84)	0.88
CCI	5 (5)	2 (6)	< 0.01*	6 (4)	2 (5)	< 0.01*
Blood markers						
Albumin (g/dL)	3 (1.10)	3.2 (0.88)	0.03*	3.1 (0.70)	3.3 (1.10)	0.13
Hb (g/dL)	10 (4.10)	12.2 (4.50)	< 0.01*	10.1 (3.93)	12 (4.90)	0.07
PLT (× 10^3^/µL)	205 (104)	192 (129)	0.42	154.5 (122)	166 (134)	0.96
Cr (mg/dL)	3.24 (4.61)	1.24 (0.72)	< 0.01*	5.95 (6.40)	1.62 (1.41)	< 0.01*
Na (mEq/L)	137 (8)	140 (7)	0.01*	139.5 (8.75)	141 (9.75)	0.24
K (mEq/L)	4.85 (1.43)	4.50 (1.90)	0.07	5.50 (3.17)	6.10 (2.80)	0.03*
CK (U/L)	208.5 (485)	183 (276.5)	0.3	182.5 (428.25)	218 (395)	0.98
Troponin I (ng/mL)	0.71 (1.33)	0.41 (1.42)	0.09	0.43 (2.13)	0.32 (0.87)	0.50
PH	7.19 (0.22)	7.16 (0.36)	0.69	7.04 (0.19)	6.96 (0.27)	0.02*
PCO2 (mmHg)	44.8 (28.55)	54.3 (39.7)	0.01*	78.7 (50)	73.6 (43.60)	0.74
Lactate (mmol/L)	91.2 (82.60)	74.75 (84.42)	0.2	96.45 (65.65)	131.7 (79.80)	0.02*
Ca (mg/dL)	8.60 (1.40)	8.40 (1.50)	0.08	9.25 (1.45)	8.90 (1.80)	0.42
ED treatment						
Electric Shock (n, %)	16 (13.22)	42 (6.33)	0.01*	7 (25)	53 (13.15)	0.14
Epinephrine (amp)	0 (5)	0 (3.50)	0.80	9.5 (6.25)	10 (6)	0.19
7% NaHCO3 (amp)	8 (7.50)	6 (6)	< 0.01*	8 (5.75)	6 (6)	0.18
Amiodarone (amp)	0 (0)	0 (0)	0.12	0 (0)	0 (0)	0.03*
Lidocaine (amp)	0 (0)	0 (0)	0.81	0 (0)	0 (0)	0.43
CPR duration in ED (minute)	1.5 (13.50)	1.5 (9)	0.8	28.5 (18.75)	30 (18)	0.19
Sustained ROSC (n, %)	90 (74.38)	199 (30.02)	< 0.01*			
Length of ED stay (Hour)	5.65 (7.11)	1.87 (2.79)	< 0.01*	2.84 (3.85)	2.2 (2.01)	0.31
Length of hospital stay (Day)	13.37 (27.49)	12.27 (24.47)	0.38			
Survival discharge (n, %)	35 (38.89)	90 (45.23)	0.38			

*ROSC* return of spontaneous circulation, *ESKD* end-stage kidney disease, *IQR* interquartile range, *COPD* chronic obstructive pulmonary disease, *CLD* chronic liver disease, *CKD* chronic kidney disease, *ED* emergency department, *PAOD* peripheral artery occlusive disease, *IHD* ischemic heart disease, *Hb* hemoglobin, *PCO2* partial pressure of carbon dioxide, *NaHCO3* sodium bicarbonate.

*P < 0.05.

Among the blood markers, patients with any ROSC in the ESKD group had lower potassium levels (median, IQR: 5.5, 1.41 mEq/L) than those in the non-ESKD group (5.91, 0.81 mEq/L), *P* < 0.01. They also had higher pH levels (median, IQR: 7.1, 0.19 vs. 7.06, 0.02, *P* < 0.01). Lactate levels (111.8, 50.6 mmol/L) were of no difference compared to the non-ESKD group (111.8, 3.92 mmol/L), *P* = 0.20. The patients without any ROSC in the ESKD group had lower potassium levels (median, IQR: 5.5, 3.17 mEq/L) than those in the non-ESKD group (6.1, 2.80 mEq/L), *P* = 0.03. They also had higher pH levels (median, IQR: 7.04, 0.18 vs. 6.96, 0.26, *P* = 0.02). Lactate levels (99.86, 55.23 mmol/L) were lower compared to the non-ESKD group (121.5, 65.73 mmol/L), *P* = 0.02.

As for emergency treatment procedures, the ESKD group with ROSC received more electric shocks (13.22%) and sodium bicarbonate infusions (median: 4 ampules, 1 ampule = 20 mL = 16.6 mEq HCO3−) than the non-ESKD group with ROSC (median 0 ampules), *P* < 0.01. At the same time, the ESKD group without ROSC received more sodium bicarbonate infusions (median, IQR: 7, 5.5 ampules) than the non-ESKD group without ROSC (4, 8 ampules), *P* < 0.01. Similar results were observed in the post PSM analyses (Table S1-A, B).

### Effect of ESKD on ROSC

Unadjusted logistic regression analyses reported ESKD to be positively associated with any ROSC in OHCA patients (crude odds ratio [OR]: 2.63, 95% CI 1.74–4.11, *P* < 0.01). ESKD was again significantly associated with any ROSC after adjusting all clinically important variables (adjusted OR: 4.82, 95% CI 2.70–8.95, *P* < 0.01) (Table 2). Addition, ESKD was significantly associated with sustained ROSC (adjusted OR: 7.54, 95% CI 3.20–20.89, *P* < 0.01) (Table 3).

**Table 2:** Logistic regression analysis of “any-ROSC” associated with ESKD and other variables in OHCA patients.

	Unadjusted model			^†^Adjusted model		
	OR	95% CI	*P* value	Adjusted OR	95% CI	*P* value
ESKD (Yes)	2.63	1.74, 4.11	< 0.01*	4.82	2.70, 8.95	< 0.01*
Sex (Male)	0.95	0.74, 1.22	0.70	0.92	0.67, 1.26	0.59
Age	0.99	0.99, 1.00	0.03*	0.99	0.98, 1	< 0.01*
Baseline comorbidities						
HTN	0.97	0.72, 1.31	0.82	0.81	0.53, 1.23	0.32
DM	1.05	0.81, 1.37	0.70	1.03	0.73, 1.46	0.85
COPD	1.06	0.81, 1.39	0.69	1.04	0.72, 1.52	0.83
CLD	1.52	0.96, 2.48	0.09	1.32	0.75, 2.38	0.34
CKD	0.84	0.64, 1.12	0.24	1.32	0.89, 1.95	0.16
PAOD	0.64	0.38, 1.08	0.09	0.54	0.28, 1.04	0.06
Stroke	0.94	0.73, 1.21	0.62	0.78	0.55, 1.10	0.16
IHD	1.04	0.74, 1.49	0.81	1.15	0.73, 1.82	0.56
Cancer	1.55	1.18, 2.05	< 0.01*	1.10	0.79, 1.55	0.57
Blood markers						
Albumin	1.01	0.74, 1.39	0.94	1.31	0.87, 2	0.20
Hb	0.96	0.92, 1.01	0.11	0.95	0.89, 1.01	0.11
PLT	1	1, 1	< 0.01*	1	1, 1	0.29
Creatinine	0.95	0.89, 1	0.06	0.94	0.86, 1.03	0.20
Na	0.99	0.98, 1	0.31	1	0.98, 1.01	0.83
K	0.73	0.67, 0.78	< 0.01*	0.82	0.75, 0.9	< 0.01*
CK	1	1, 1	0.46	1	1, 1	0.43
Troponin I	1	0.99, 1.02	0.96	1	0.98, 1.02	0.76
PH	56.81	27.37, 122.5	< 0.01*	6.48	2.01, 21.13	< 0.01*
PCO2	0.98	0.98, 0.99	< 0.01*	0.99	0.99, 1	0.06
Lactate	0.99	0.98, 0.99	< 0.01*	1	0.99, 1	0.03*
Ca	0.82	0.75, 0.91	< 0.01*	0.97	0.86, 1.09	0.58
ED treatment						
Electric shock	0.49	0.34, 0.72	< 0.01*	0.80	0.44, 1.46	0.47
7% NaHCO3	0.20	0.16, 0.26	< 0.01*	0.17	0.13, 0.24	< 0.01*
Amiodarone	0.40	0.25, 0.65	< 0.01*	0.47	0.23, 0.97	0.04*
Lidocaine	0.48	0.18, 1.27	0.14	1.05	0.31, 3.42	0.94

*ROSC* return of spontaneous circulation, *ESKD* end-stage kidney disease, *COPD* chronic obstructive pulmonary disease, *CLD* chronic liver disease, *CKD* chronic kidney disease, *ED* emergency department, *PAOD* peripheral artery occlusive disease, *IHD* ischemic heart disease, *Hb* hemoglobin, *PCO2* partial pressure of carbon dioxide, *NaHCO3* sodium bicarbonate.

^**†**^Adjusted for sex, age, baseline comorbidities, all blood markers, and ED treatments. *P value < 0.05.

**Table 3:** Logistic regression analysis of “sustained ROSC” associated with ESKD and other variables in OHCA patients.

	Unadjusted model			^†^ Adjusted model		
	OR	95% CI	*P* value	Adjusted OR	95% CI	*P* value
ESKD (Yes)	6.77	4.4, 10.66	< 0.01*	9.45	3.83, 24.13	< 0.01*
Sex (Male)	1.02	0.75, 1.39	0.92	1.34	0.71, 2.56	0.37
Age	0.99	0.98, 0.99	< 0.01*	0.98	0.96, 0.99	< 0.01*
Baseline comorbidities						
HTN	1.06	0.73, 1.53	0.77	0.42	0.17, 1.01	0.06
DM	0.99	0.72, 1.35	0.93	0.46	0.22, 0.93	0.03*
COPD	0.58	0.41, 0.82	< 0.01*	1.33	0.62, 2.84	0.46
CLD	0.89	0.52, 1.49	0.65	1.43	0.53, 3.67	0.47
CKD	0.43	0.29, 0.64	< 0.01*	1.11	0.48, 2.53	0.80
PAOD	1.03	0.48, 2.11	0.94	1.05	0.29, 3.47	0.94
Stroke	0.89	0.64, 1.23	0.48	0.77	0.37, 1.57	0.47
IHD	3.26	2.13, 5.04	< 0.01*	4.02	1.70, 9.76	< 0.01*
Cancer	0.48	0.34, 0.66	< 0.01*	0.66	0.33, 1.29	0.24
Blood markers						
Albumin	0.98	0.61, 1.57	0.93	0.77	0.27, 2.15	0.62
Hb	0.96	0.89, 1.03	0.22	0.85	0.72, 0.99	0.05*
PLT	1.01	1, 1.01	< 0.01*	1.01	1, 1.01	< 0.01*
Creatinine	1.05	0.95, 1.15	0.32	1.28	1.01, 1.67	0.05*
Na	0.97	0.94, 0.99	0.02*	0.94	0.90, 0.97	< 0.01*
K	0.16	0.12, 0.21	< 0.01*	0.16	0.11, 0.24	< 0.01*
CK	1	1, 1	0.01*	1	1, 1	0.42
Troponin I	0.83	0.76, 0.90	< 0.01*	0.91	0.84, 0.98	0.01*
PH	15,123.23	3121, 85,308	< 0.01*	1024.83	36, 31,201	< 0.01*
PCO2	0.94	0.93, 0.95	< 0.01*	1	0.98, 1.01	0.69
Lactate	0.98	0.97, 0.98	< 0.01*	1	0.99, 1	0.35
Ca	0.85	0.75, 0.97	0.02*	1.12	0.88, 1.44	0.36
ED treatment						
Electric shock	4.26	2.43, 7.75	< 0.01*	1.49	0.44, 5.20	0.53
7% NaHCO3	5.22	3.76, 7.30	< 0.01*	10.79	5.81, 20.69	< 0.01*
Amiodarone	5.51	2.55, 13.26	< 0.01*	2.20	0.46, 11.74	0.34
Lidocaine	5.23	1.20, 35.83	0.04*	0.30	0.03, 3.74	0.33

*ROSC* return of spontaneous circulation, *ESKD* end-stage kidney disease, *COPD* chronic obstructive pulmonary disease, *CLD* chronic liver disease, *CKD* chronic kidney disease, *ED* emergency department, *PAOD* peripheral artery occlusive disease, *IHD* ischemic heart disease, *Hb* hemoglobin, *PCO2* partial pressure of carbon dioxide, *NaHCO3* sodium bicarbonate.

^**†**^Adjusted for sex, age, baseline comorbidities, all blood markers, and ED treatments. *P value < 0.05.

After PS matching by age, sex, and baseline comorbidities, in a 1:4 ratio, ESKD still demonstrated to be positively associated with any ROSC (adjust OR: 4.92, 95% CI 2.71–9.35, *P* < 0.01) (Table S2) and sustained ROSC (adjusted OR: 5.15, 95% CI 2.45–10.96, *P* < 0.01) (Table S3) after adjusting for clinically important variables. Subgroup analysis which subdivided the OHCA patients into non-CKD, CKD, and ESKD groups demonstrated that ESKD remained to be positively associated with any ROSC (adjusted OR: 4.82, 95% CI 2.70–8.95, *P* < 0.01) (Table S4) and sustained ROSC (adjusted OR: 9.45, 95% CI 3.83–24.13, *P* < 0.01) (Table S5).

### Survival analysis

**Figure 2:**
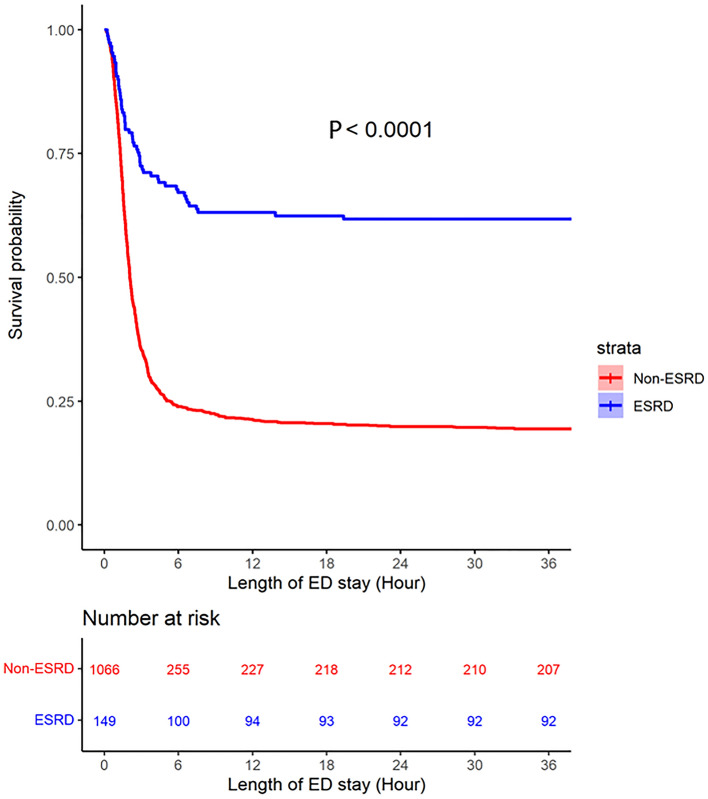
Kaplan–Meier survival curve used to evaluate the effect of end-stage kidney disease (ESKD) on the rate of attaining sustained return of spontaneous circulation (ROSC) in patients with out-of-hospital cardiac arrest (OHCA) for the evaluated time point, emergency department (ED) stay (survival until hospital admission). Patients with ESKD (blue). Patients without ESKD or CKD (red). The x-axis shows the time in hours of ED stay, and the y-axis shows the sustained survival probability of OHCA patients with cardio-pulmonary resuscitation (CPR).

Figure 2 provides a Kaplan–Meier plot of ED survival resulting from sustained ROSC (or called “survival event”) in OHCA patients with and without ESKD for the censored time point, that is, surviving until hospital admission. The ESKD group had a higher probability of achieving sustained ROSC than the non-ESKD group. The plot started from the ED arrival time of every OHCA patient receiving CPR. The survival probability of the non-ESKD group from hour 0 to hour 4 dropped to approximately 30%, but almost 75% of the ESKD group survived. Moreover, almost 60% of the ESKD group survived to admission. The log-rank tests further confirmed that ESKD was significantly associated with the “sustained ROSC” probability (*P* < 0.01) until hospital admission in patients with OHCA.

**Figure 3:**
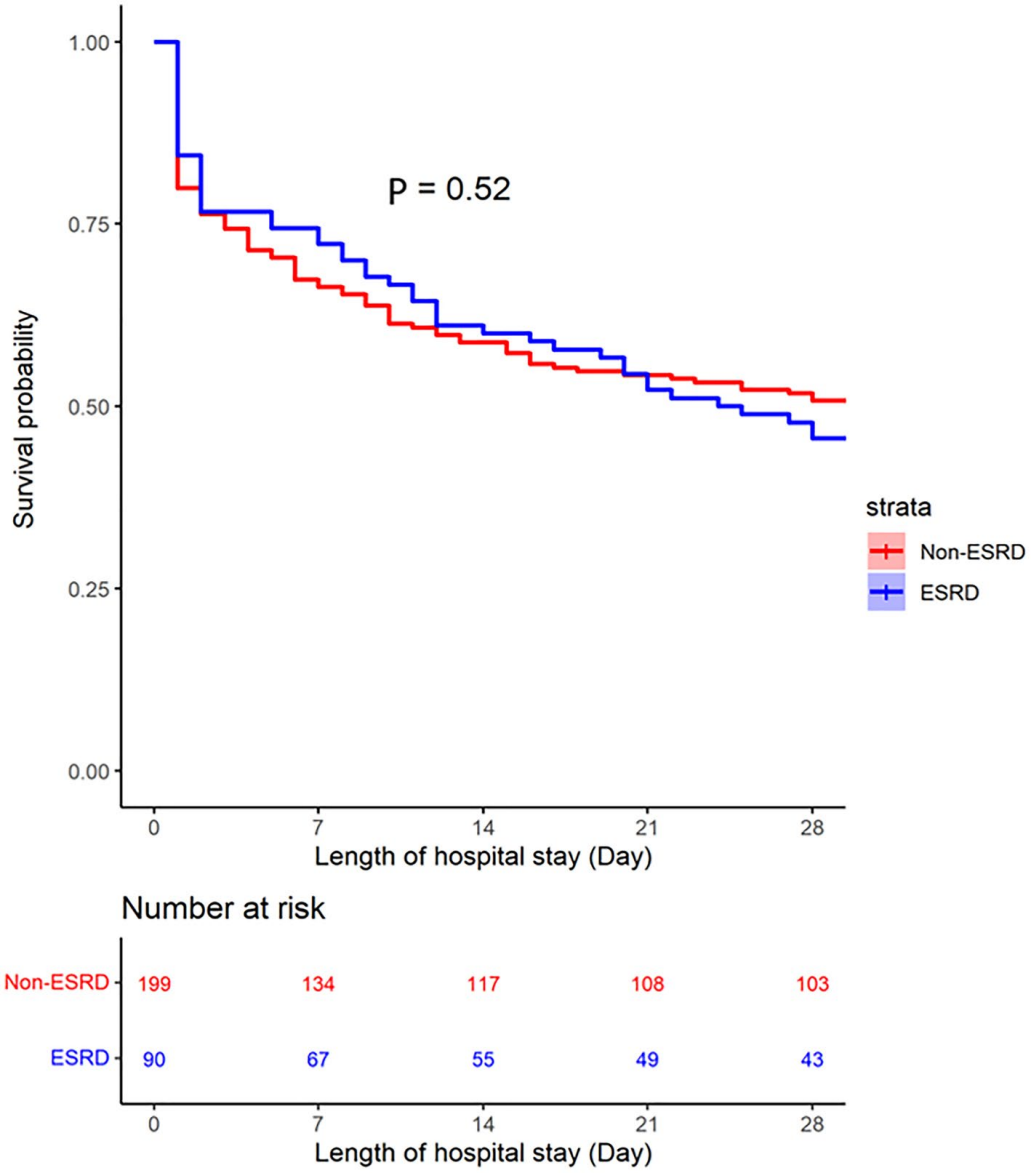
Kaplan–Meier survival curve used to evaluate the effect of end-stage kidney disease (ESKD) on the 28-day hospital survival in patients with sustained return of spontaneous circulation (ROSC). Patients with ESKD (blue). Patients without ESKD (red). The x-axis depicts the number of days of hospital stay, and the y-axis shows the hospital survival probability.

Figure 3 similarly provides the Kaplan–Meier plot of hospital survival in patients with and without ESKD for evaluating hospital stay duration, which was censored until 28 days. The 28 day-survival between the OHCA patients with and without ESKD showed no differences. Figures S1 and S2 demonstrates similar results after dividing the non-ESKD patients into non-CKD and CKD subgroups.

## Discussion

OHCA with poor outcomes is one of the several global health issues whose incidence has increased over the years because of the increased life expectancy of populations worldwide. Approximately 60% of patients with OHCA die without sustained ROSC, and 60–80% may die in ICUs after revival in the ED^21^. ED physicians commonly observe in their clinical practice that patients with ESKD have a higher rate of ROSC in CPR than the general population. Therefore, this study included patients with ESKD undergoing regular HD between 2010 and 2020 and compared them with those without ESKD in terms of the incidence rate of ROSC after OHCA. Even though usually ESKD patients inevitably has a much shorter lifespan even after receiving dialysis therapy, than non-ESKD patients^9,22^, findings from the current study indicated that ESKD patients undergoing maintenance HD was positively associated with attaining ROSC after encountering OHCA events. It conferred more than four-times the odds of attaining any ROSC and nine-times the odds of sustained-ROSC when compared to patients without ESKD. Moreover, the ESKD patients had lower serum potassium levels than their counterparts. Based on the evidence, it is suggested that (1) ESKD patients in OHCA should not be treated under the stereotypical assumption of hyperkalemia or severe acidosis, contrary to the ACLS, 5H5T protocol (5H: hypoxia, hypovolemia, hypothermia, hydrogen ion (acidosis), and hyper- or hypokalemia).

According to our prior study, the incidence of OHCA in ESKD patients undergoing maintenance HD was much higher than that in the general population. In the study, we also found ESKD patients undergoing maintenance HD had a higher probability of attaining ROSC. To investigate the insidious causal-relationship or mechanism, this hospital-based study including real-world data was conducted. This study provides newer insights into the possibilities of ROSC for OHCA patients with ESKD undergoing maintenance HD. Based on this, it is also suggested (2) to disregard the futility of extended CPRs on ESKD patients as the efforts will not be wasted due to the proven higher rates of ROSC through this study.

This study is the first to reverse the stereotypical knowledge that (1) patients with ESKD who experienced OHCA have a lower ROSC rate because of the multiple cardiovascular burdens, so continuing resuscitation is often ceased, and that (2) they have a higher incidence rate of hyperkalemia and more severe acidosis than those without ESKD. Usually, more sodium bicarbonate infusions are ad-ministered to correct hyperkalemia and acidosis, which is a common scenario in every ED, however, one of the major findings of this cohort study is that not all patients with ESKD undergoing HD have more severe hyperkalemia and acidosis.

ESKD, being positively associated with ROSC in OHCA patients, could be explained by the training or improvement of cardiovascular compliance and tolerance to toxins during regular HD when the blood is drawn away from the body and is circulated back into it again. Every organ acquires tolerance and gets accustomed to hyperkalemia and severe acidosis, which are generally induced by repeated ischemia–reperfusion injury^23,24^. Considering that our hypothesis was built mainly on this premise, those receiving PD were excluded from this study.

In the clinical practice, ESKD patients frequently had syncope because of low blood volume after HD. And these patients usually had good prognosis when CPR was performed on time. However, the study subjects of ESKD in our study were quite different; they were mostly sent to our ED from the scene of OHCA occurrence, such as residence area or public space, rather than from the affiliated HD centers of our hospital or the neighboring HD clinic. Therefore, this group of ESKD patients with OHCA may share only a part of the mechanism as mentioned above (low blood volume and syncope). In conclusion, the location of arrest was an important confounding factor as arrests in residential areas versus public spaces and high-rise settings were invariably associated with poorer OHCA outcomes^25,26^.

Previous studies hypothesized an influence on arterial hemodynamics via blood volume and the vasoactive hormonal profile change during an HD session^23^. HD allowed the removal of vasoconstricting factors and induction of vasodilating factors. Another hypothesis was that alkaline compounds (e.g., sodium bicarbonate, tris-hydroxymethyl aminomethane, and tribunate), which had been traditionally used during HD, acted as treatment regimens for metabolic acidosis^27^. However, sodium bicarbonate administration during CPR remained controversial because of its inconsistent benefits^28–30^. It induced intracellular acidosis, thereby potentially caused adverse outcomes. Given that findings from this study demonstrated that patients treated with higher doses of sodium carbonate seemed to have higher odds of sustained ROSC, we should assess whether sodium bicarbonate administration was beneficial during the resuscitation of patients with ESKD.

The outcomes of OHCA care, involving invasive managements, particularly in terms of coronary angiography, coronary revascularization, and mechanical support, may vary^31–33^. Therapeutic hypothermia has emerged as a promising treatment in recent years for improving outcomes in OHCA patients^34–36^. There are many confounding variables that can affect both the cause and effect of OHCA^37,38^. For instance, differences in patient outcomes can be attributed to the management received at the emergency department following OHCA. Among the total of 1215 patients in this study, there were 64 patients receiving coronary angiography, 38 ones receiving hypothermia therapy, and 23 ones receiving ECMO, respectively. The “chain-of-survival” concept for resuscitation of OHCA patients has evolved over the years, and patient survival depends on the collective efforts of bystanders, first responders, emergency medical services, various emergency physicians and their teams. PS matching was performed to pseudo-randomize the ESKD and non-ESKD patients with OHCA to eliminate these unknown potential confounding factors.

### Strengths and limitations

This study is the first to support the hypothesis that ESKD patients undergoing maintenance HD potentially has a positive effect on OHCA by conferring a higher OR of revival (ROSC). This study is also the first to compare the potassium and pH levels be-tween patients with and without ESKD during OHCA in Taiwan. These data may at least help refine the current management of ESKD in this country, considering that our findings totally reversed our previous idea on electrolytes in ESKD patients with OHCA. Given that the database was retrieved from one of the largest medical centers in Taiwan, its credibility was substantially more convincing.

We did not discuss the pre-ED process (e.g., the time of arrival and departure from the scene, the time the patient arrived at the ED, the duration of pre-ED CPR (such as bystander CPR and witness CPR), and the initial rhythm) because it could influence the outcomes. We could not totally exclude the potential impact of bystander CPR for patients with chronic illness, such as, ESKD, where they were more likely to have someone nearby to help perform pre-hospital management and call for help. The location of arrest was also an important confounding factor as arrests in residential areas versus public spaces and high-rise settings were invariably associated with poorer OHCA outcomes. This information was unavailable in the hospital-based database. Nevertheless, we had eliminated a part of the bystander CPR bias by excluding ESKD patients, who encountered OHCA in HD clinics under the monitoring of medical personnel, from these analyses. Finally, due to the observational nature of the study, external validation may be required, and extrapolation to other populations may not be feasible at this time.

## Conclusion

The patients with ESKD who experienced OHCA do not have higher serum potassium levels and more severe acidosis compared with the general population in Taiwan; therefore, we should not treat them with the assumption that hyperkalemia and acidosis always occur. The hypothesis of this study and the related supporting findings, are novel and important to be shared with the emergency medicine practitioners as the phenomenon of ESKD patients on HD achieving ROSC with better odds is observed by physicians quite often in the ED.

## Supplementary Information

Supplementary Information.

## Data Availability

The data that support the findings of this study are available from Taichung veteran’s general hospital, but restrictions apply to the availability of these data, which were used under license for the current study, and so are not publicly available. Data are however available from the authors upon reasonable request and with permission of Taichung veteran’s general hospital, Taiwan.
