# Supplementary material for: Effect of end-stage kidney disease on the return of spontaneous circulation in Taiwanese adults with out-of-hospital cardiac arrest
Source: Sci Rep. 2023 May 16;13:7905. doi: 10.1038/s41598-023-35024-8 (PMC10188509; doi:10.1038/s41598-023-35024-8)
Supplement: Supplementary file 1 — Supplementary Information. [file 41598_2023_35024_MOESM1_ESM.pdf]

Supplementary Materials

Title: ***Effect of end-stage kidney disease on the return of spontaneous circulation in Taiwanese adults with out-of-hospital cardiac arrest***

Authors: Ming-Shun Hsieh, MD, PhD[1-4]. Amrita Chattopadhyay, PhD[5]. Tzu-Pin Lu, PhD[6]. Shu-Hui Liao, MS[7]. Chia-Ming Chang, MD[2-3, 8]. Yi-Chen Lee, MD[1]. Wei-En Lo, BSc[1]. Jia-Jun Wu, MD[9]. Vivian Chia-Rong Hsieh[10]. Sung-Yuan Hu, MD, PhD[4, 11-13#]. Chorng-Kuang How, MD, PhD[2-3#].

Affiliations:

[1]Department of Emergency Medicine, Taipei Veterans General Hospital, Taoyuan Branch, Taoyuan, Taiwan.

[2]Department of Emergency Medicine, Taipei Veterans General Hospital, Taipei, Taiwan.

[3]School of Medicine, National Yang Ming Chiao Tung University, Taipei, Taiwan.

[4]Department of Emergency Medicine, Taichung Veterans General Hospital, Taichung, Taiwan.

[5] Bioinformatics and Biostatistics Core, Center of Genomics and Precision Medicine, National Taiwan University, Taipei, Taiwan.

[6]Department of Public Health, National Taiwan University, Taipei, Taiwan.

[7]Department of Pathology and Laboratory, Taipei Veterans General Hospital, Taoyuan Branch, Taoyuan, Taiwan.

[8]Institute of Occupational Medicine and Industrial Hygiene, National Taiwan University College of Public Health, Taipei, Taiwan;

[9]Department of Critical Care Medicine, Taipei Veterans General Hospital, Taoyuan Branch, Taoyuan, Taiwan;

[10]Department of Health Services Administration, China Medical University, Taichung, Taiwan.

[11]School of Medicine, Chung Shan Medical University, Taichung, Taiwan.

[12]Institute of Medicine, Chung Shan Medical University, Taichung, Taiwan.

[13]Department of Post-Baccalaureate Medicine, College of Medicine, National Chung Hsing University, Taichung, Taiwan.

#The two authors contributed equally to this work.

***Corresponding author (for all communication): Chorng-Kuang How,** MD, PhD, Department of Emergency Medicine, Taipei Veterans General Hospital, Taipei, Taiwan; School of Medicine, National Yang Ming Chiao Tung University, Taipei, Taiwan.

E-mail: ckhow@vghtpe.gov.tw

Table S1A. Descriptive statistics of all out-of-hospital cardiac arrest (OHCA) patients with and without end-stage kidney disease (ESKD) after 1:4 PS matching by sex, age, and baseline comorbidities.

**Variables**	**Out-of-hospital cardiac arrest (OHCA) patients with resuscitation (n = 745)**		
	ESKD = Yes (n = 149)	Non-ESKD (n = 596)	P-value
Sex			0.71
Male	99 (66.44)	408 (68.46)	
Female	50 (33.56)	188 (31.54)	
Age (years)			< 0.01*
20-29	3 (2.11)	42 (7.54)	
30-39	5 (3.52)	38 (6.82)	
40-49	12 (8.45)	47 (8.44)	
50-59	27 (19.01)	68 (12.21)	
60-69	35 (24.65)	94 (16.88)	
70-85	47 (33.10)	178 (31.96)	
≥ 85	13 (9.15)	90 (16.16)	
Median (IQR)	67.09 (17.80)	66.59 (35.20)	0.90
Baseline comorbidities			
HTN	61 (40.94)	153 (25.67)	< 0.01*
DM	76 (51.01)	233 (39.09)	0.01*
Hyperlipidemia	30 (20.13)	54 (9.06)	< 0.01*

COPD	39 (26.17)	139 (23.32)	0.53
CLD	21 (14.09)	62 (10.40)	0.26
CKD	0 (0)	178 (29.87)	< 0.01*
PAOD	16 (10.74)	30 (5.03)	0.02*
Stroke	55 (36.91)	176 (29.53)	0.10
IHD	40 (26.85)	96 (16.11)	< 0.01*
Cancer	37 (24.83)	143 (23.99)	0.91
CCI	5 (5)	3 (6)	< 0.01*
Blood markers†			
Albumin (g/dL)	3.13 (0.34)	3.13 (0.13)	0.06
Hb (g/dL)	11.4 (2.70)	11.67 (2.53)	< 0.01*
PLT (x10^3/μL)	196.53 (84)	196.53 (47)	0.09
Cr (mg/dL)	2.45 (3.79)	2.45 (1.18)	< 0.01*
Na (mEq/L)	139.88 (6)	139.88 (3)	< 0.01*
K (mEq/L)	5.5 (1.51)	5.91 (1.01)	< 0.01*
CK (U/L)	353 (633.37)	725.73 (562.87)	0.01*
Troponin I (ng/mL)	1.57 (3.13)	3.56 (2.98)	0.20
PH	7.09 (0.18)	7.06 (0.12)	< 0.01*
PCO2 (mmHg)	61.8 (29.6)	69.2 (17.33)	< 0.01*
Lactate (mmol/L)	111.8 (53.58)	111.8 (20.62)	0.01*
Ca (mg/dL)	8.42 (0.85)	8.42 (0.50)	0.46

ED treatment			
Electric Shock (n, %)	23 (15.44)	66 (11.07)	0.18
Epinephrine (amp) †	1 (6)	4 (10)	< 0.01*
7% NaHCO3 (amp) †	5 (9)	0 (5)	< 0.01*
Amiodarone (amp) †	0 (0)	0 (0)	0.13
Lidocaine (amp) †	0 (0)	0 (0)	0.26
CPR duration in ED (minute)	3 (16.5)	12 (28.5)	< 0.01*
Sustained ROSC (n, %)	90 (74.38)	126 (33.96)	< 0.01*
Length of ED stay (Hour) †	4.65 (5.84)	2.05 (2.55)	< 0.01*
Length of hospital stay (Day) †	13.37 (27.49)	12.27 (26.62)	0.72
Survival discharge (n, %)	35 (38.89)	61 (48.41)	0.21
Coronary angiography	13 (9.03)	36 (6.05)	0.27

ROSC: return of spontaneous circulation; ESKD: end-stage kidney disease; IQR: interquartile range; COPD: chronic obstructive pulmonary disease; CLD: chronic liver disease; CKD: chronic kidney disease; ED: emergency department; PAOD: peripheral artery occlusive disease; IHD: ischemic heart disease; Hb: hemoglobin; PCO2: partial pressure of carbon dioxide; NaHCO3: sodium bicarbonate. * P < 0.05. † Median (IQR).

Table S1B. Descriptive statistics of all out-of-hospital cardiac arrest (OHCA) patients with and without end-stage kidney disease (ESKD) after 1:4 PS matching by sex, age, and baseline comorbidities.

**Variables**	**Out-of-hospital cardiac arrest (OHCA) patients with resuscitation (n = 745)**					
	**Any ROSC=Yes (n = 492)**			**Any ROSC=No (n = 253)**		
	ESKD = Yes (n = 121)	Non-ESKD (n = 371)	P-value	ESKD = Yes (n = 28)	Non-ESKD (n = 225)	P-value
Sex			1			0.51
Male	82 (67.77)	253 (68.19)		17 (60.71)	155 (68.89)	
Female	39 (32.23)	118 (31.81)		11 (39.29)	70 (31.11)	
Age (years)			< 0.01*			0.83
20-29	2 (1.75)	32 (9.41)		1 (3.57)	10 (4.61)	
30-39	3 (2.63)	23 (6.76)		2 (7.14)	15 (6.91)	
40-49	11 (9.65)	29 (8.53)		1 (3.57)	18 (8.29)	
50-59	21 (18.42)	43 (12.65)		6 (21.43)	25 (11.52)	
60-69	31 (27.19)	58 (17.06)		4 (14.29)	36 (16.59)	
70-85	36 (31.58)	98 (28.82)		11 (39.29)	80 (36.87)	

≥ 85	10 (8.77)	57 (16.76)		3 (10.71)	33 (15.21)	
Median (IQR)	66.69 (17.46)	65.32 (39.15)	0.66	69.67 (19.74)	70.15 (28.1)	0.88
Baseline comorbidities						
HTN	46 (38.02)	90 (24.26)	< 0.01*	15 (53.57)	63 (28)	0.01*
DM	60 (49.59)	143 (38.54)	0.04*	16 (57.14)	90 (40)	0.13
Hyperlipidemia	30 (24.79)	53 (14.29)	0.01*	0 (0)	1 (0.44)	1
COPD	28 (23.14)	87 (23.45)	1	11 (39.29)	52 (23.11)	0.10
CLD	17 (14.05)	45 (12.13)	0.69	4 (14.29)	17 (7.56)	0.27
CKD	-	106 (28.57)	-	-	72 (32)	-
PAOD	10 (8.26)	14 (3.77)	0.08	6 (21.43)	16 (7.11)	0.02*
Stroke	45 (37.19)	109 (29.38)	0.13	10 (35.71)	67 (29.78)	0.67
IHD	31 (25.62)	58 (15.63)	0.02*	9 (32.14)	38 (16.89)	0.09
Cancer	30 (24.79)	103 (27.76)	0.60	7 (25)	40 (17.78)	0.50
CCI	5 (5)	3 (5)	< 0.01*	6 (4)	2 (5)	< 0.01*
Blood markers†						

Albumin (g/dL)	3.13 (0.24)	3.13 (0)	0.24	3.11 (0.50)	3.16 (0.35)	0.13
Hb (g/dL)	11.6 (2.77)	11.67 (0.03)	< 0.01*	10.1 (3.93)	12.49 (4.50)	0.02*
PLT ($x10^3/\mu L$)	196.53 (76)	196.53 (0)	0.43	154.5 (122)	161 (119)	0.85
Cr (mg/dL)	2.45 (2.60)	2.45 (1.07)	< 0.01*	5.95 (6.40)	1.62 (1.32)	< 0.01*
Na (mEq/L)	139.88 (6)	139.88 (0)	0.01*	139.5 (8.75)	140 (9)	0.33
K (mEq/L)	5.5 (1.41)	5.91 (1.11)	0.03*	5.5 (3.17)	6 (2.7)	0.04*
CK (U/L)	500 (612.37)	753.37 (206.28)	< 0.01*	182.5 (428.25)	238 (445)	0.60
Troponin I (ng/mL)	1.93 (3.03)	3.58 (1.90)	< 0.01*	0.69 (1.51)	0.73 (2.13)	0.94
PH	7.1 (0.19)	7.06 (0.06)	< 0.01*	7.04 (0.18)	6.97 (0.26)	0.02*
PCO2 (mmHg)	56.8 (29.90)	69.2 (8.55)	< 0.01*	76.45 (48.45)	76.08 (40.20)	0.40
Lactate (mmol/L)	111.8 (50.60)	111.8 (9.89)	0.40	99.86 (55.23)	117.7 (55.9)	0.05
Ca (mg/dL)	8.42 (0.80)	8.42 (0)	0.19	8.75 (1.34)	8.7 (1.64)	0.36
ED treatment						
Electric Shock (n, %)	16 (13.22)	30 (8.09)	0.13	7 (25)	36 (16)	0.28
Epinephrine (amp) †	0 (5)	0 (4)	0.99	9.5 (6.25)	10 (7)	0.13

7% NaHCO3 (amp) †	4 (9)	0 (0)	< 0.01*	7 (5.50)	4 (8)	< 0.01*
Amiodarone (amp) †	0 (0)	0 (0)	0.29	0 (0)	0 (0)	0.04*
Lidocaine (amp) †	0 (0)	0 (0)	0.52	0 (0)	0 (0)	0.39
CPR duration in ED (minute)	1.5 (13.50)	1.5 (10.50)	0.99	28.5 (18.75)	30 (21)	0.13
Sustained ROSC (n, %)	90 (74.38)	126 (33.96)	< 0.01*			
Length of ED stay (Hour) †	5.65 (7.11)	1.92 (2.86)	< 0.01*	2.84 (3.85)	2.21 (2.21)	0.44
Length of hospital stay (Day) †	13.37 (27.49)	12.27 (26.62)	0.72			
Survival discharge (n, %)	35 (38.89)	61 (48.41)	0.21			
Coronary angiography	12 (10.08)	35 (9.46)	0.98	1 (4)	1 (0.44)	0.19

ROSC: return of spontaneous circulation; ESKD: end-stage kidney disease; IQR: interquartile range; COPD: chronic obstructive pulmonary disease; CLD: chronic liver disease; CKD: chronic kidney disease; ED: emergency department; PAOD: peripheral artery occlusive disease; IHD: ischemic heart disease; Hb: hemoglobin; PCO2: partial pressure of carbon dioxide; NaHCO3: sodium bicarbonate. * $P < 0.05$. † Median (IQR).

Table S2. Logistic regression analysis of "any-ROSC" associated with ESKD and other variables in OHCA patients after 1:4 PS matching by sex, age, and baseline comorbidities..

	Unadjusted model			† Adjusted model		
	**OR**	**95% CI**	**P-value**	**Adjusted OR**	**95% CI**	**P-value**
ESKD (Yes)	2.62	1.71, 4.15	< 0.01*	4.92	2.71, 9.35	< 0.01*
Blood markers						
Albumin	1.01	0.69, 1.48	0.94	1.68	1.03, 2.77	0.04*
Hb	0.92	0.86, 0.98	< 0.01*	0.90	0.84, 0.97	< 0.01*
PLT	1	1, 1.01	< 0.01*	1	1, 1.01	< 0.01*
Creatinine	0.96	0.90, 1.03	0.29	0.90	0.81, 0.99	0.04*
Na	1	0.99, 1.01	0.59	1.01	0.99, 1.02	0.56
K	0.77	0.70, 0.83	< 0.01*	0.86	0.76, 0.96	< 0.01*
CK	1	1, 1	0.36	1	1, 1	0.31
Troponin I	1	0.98, 1.03	0.85	0.99	0.97, 1.02	0.62
PH	50.68	20.26, 134.57	< 0.01*	4.37	1, 19.13	0.05*
PCO2	0.98	0.97, 0.98	< 0.01*	0.99	0.98, 1	< 0.01*
Lactate	0.99	0.99, 0.99	< 0.01*	1	0.99, 1	0.94
Ca	0.85	0.75, 0.95	< 0.01*	0.89	0.78, 1.03	0.12
ED treatment						
Electric shock	0.50	0.32, 0.79	< 0.01*	0.73	0.36, 1.49	0.38
7% NaHCO3	0.21	0.15, 0.28	< 0.01*	0.17	0.11, 0.25	< 0.01*
Amiodarone	0.45	0.25, 0.80	< 0.01*	0.60	0.25, 1.45	0.26
Lidocaine	0.59	0.20, 1.86	0.35	1.32	0.33, 5.37	0.69

ROSC: return of spontaneous circulation; ESKD: end-stage kidney disease; Hb: hemoglobin; PCO2: pressure of carbon dioxide; NaHCO3: sodium bicarbonate.

† Adjusted for age, sex, baseline comorbidities, all blood markers, and ED treatments. * P-value < 0.05.

Table S3. Logistic regression analysis of "sustained ROSC" associated with ESKD and other variables in OHCA patients after 1:4 PS matching by sex, age, and baseline comorbidities..

	Unadjusted model			† Adjusted model		
	**OR**	**95% CI**	**P-value**	**Adjusted OR**	**95% CI**	**P-value**
ESKD (Yes)	5.65	3.60, 9.07	< 0.01*	5.15	2.45, 10.96	< 0.01*
Blood markers						
Albumin	1.02	0.61, 1.71	0.93	0.64	0.20, 1.97	0.44
Hb	0.91	0.84, 0.99	0.03*	0.86	0.73, 1.01	0.08
PLT	1	1, 1.01	< 0.01*	1.01	1, 1.01	0.02*
Creatinine	1.16	1.05, 1.30	< 0.01*	1.30	1.04, 1.70	0.03*
Na	0.97	0.94, 1	0.05	0.95	0.91, 0.98	< 0.01*
K	0.2	0.14, 0.27	< 0.01*	0.15	0.09, 0.24	< 0.01*
CK	1	1, 1	0.51	1	1, 1	0.49
Troponin I	0.96	0.90, 1.01	0.14	0.94	0.86, 1.01	0.07
PH	3795.13	668.8, 26189.2	< 0.01*	315.03	7.03, 14513.45	< 0.01*
PCO2	0.95	0.94, 0.96	< 0.01*	1	0.98, 1.02	0.87
Lactate	0.98	0.98, 0.99	< 0.01*	1	0.99, 1.01	0.62
Ca	0.95	0.82, 1.10	0.5	1.05	0.77, 1.44	0.75
ED treatment						
Electric shock	4.11	2.13, 8.47	< 0.01*	2.85	0.75, 11.36	0.13
7% NaHCO3	4.60	3.08, 6.96	< 0.01*	6.75	3.38, 13.85	< 0.01*
Amiodarone	3.28	1.39, 8.63	< 0.01*	0.60	0.10, 4.08	0.59
Lidocaine	3.25	0.69, 22.82	0.16	0.51	0.05, 5.54	0.56

ROSC: return of spontaneous circulation; ESKD: end-stage kidney disease; Hb: hemoglobin; PCO2: pressure of carbon dioxide; NaHCO3: sodium bicarbonate.

† Adjusted for age, sex, baseline comorbidities, all blood markers, and ED treatments. * P-value < 0.05.

Table S4. Logistic regression analysis of "any-ROSC" associated with CKD, ESKD, and other variables in OHCA patients.

	Unadjusted model			†Adjusted model		
	**OR**	**95% CI**	**P-value**	**Adjusted OR**	**95% CI**	**P-value**
Non-CKD	Ref	-	-	Ref	-	-
CKD	0.96	0.72, 1.28	0.78	1.04	0.75, 1.45	0.82
ESKD (Yes)	2.60	1.71, 4.09	< 0.01*	2.87	1.82, 4.68	< 0.01*
Sex (Male)	0.95	0.74, 1.22	0.70	0.93	0.72, 1.21	0.60
Age	0.99	0.99, 1.00	0.03*	0.99	0.99, 1	0.03*
Baseline comorbidities						
HTN	0.97	0.72, 1.31	0.82	0.82	0.57, 1.17	0.28
DM	1.05	0.81, 1.37	0.70	0.97	0.72, 1.30	0.83
COPD	1.06	0.81, 1.39	0.69	1.19	0.86, 1.65	0.29
CLD	1.52	0.96, 2.48	0.09	1.28	0.79, 2.14	0.32
PAOD	0.64	0.38, 1.08	0.09	0.57	0.33, 1.01	0.05
Stroke	0.94	0.73, 1.21	0.62	0.96	0.72, 1.28	0.78
IHD	1.04	0.74, 1.49	0.81	1.06	0.72, 1.55	0.78
Cancer	1.55	1.18, 2.05	< 0.01*	1.65	1.24, 2.20	< 0.01*

ROSC: return of spontaneous circulation; ESKD: end-stage kidney disease; CKD: chronic kidney disease; COPD: chronic obstructive pulmonary disease; CLD: chronic liver disease; ED: emergency department; PAOD: peripheral artery occlusive disease; IHD: ischemic heart disease; Hb: hemoglobin; PCO2: pressure of carbon dioxide; NaHCO3: sodium bicarbonate.

† Adjusted for sex, age, and baseline comorbidities. * P-value < 0.05.

Table S5. Logistic regression analysis of "sustained ROSC" associated with CKD, ESKD, and other variables in OHCA patients.

	Unadjusted model			†Adjusted model		
	OR	95% CI	P-value	Adjusted OR	95% CI	P-value
Non-CKD	Ref	-	-	Ref	-	-
CKD	0.62	0.41, 0.92	0.02	0.88	0.54, 1.42	0.61
ESKD (Yes)	6.06	3.91, 9.62	< 0.01	6.65	4.04, 11.24	< 0.01*
Sex (Male)	1.02	0.75, 1.39	0.92	0.99	0.70, 1.40	0.95
Age	0.99	0.98, 0.99	< 0.01*	0.99	0.98, 1	0.03*
Baseline comorbidities						
HTN	1.06	0.73, 1.53	0.77	0.75	0.45, 1.23	0.26
DM	0.99	0.72, 1.35	0.93	0.87	0.58, 1.30	0.50
COPD	0.58	0.41, 0.82	< 0.01*	0.75	0.48, 1.16	0.20
CLD	0.89	0.52, 1.49	0.65	0.88	0.47, 1.60	0.67
PAOD	1.03	0.48, 2.11	0.94	0.85	0.35, 1.94	0.70
Stroke	0.89	0.64, 1.23	0.48	0.94	0.63, 1.40	0.77
IHD	3.26	2.13, 5.04	< 0.01*	3.59	2.20, 5.94	< 0.01*
Cancer	0.48	0.34, 0.66	< 0.01*	0.58	0.40, 0.84	< 0.01*

ROSC: return of spontaneous circulation; ESKD: end-stage kidney disease; CKD: chronic kidney disease; COPD: chronic obstructive pulmonary disease; CLD: chronic liver disease; ED: emergency department. PAOD: Peripheral artery occlusive disease; IHD: ischemic heart disease; NaHCO3: sodium bicarbonate.

†Adjusted for sex, age, and baseline comorbidities. * P-value < 0.05.

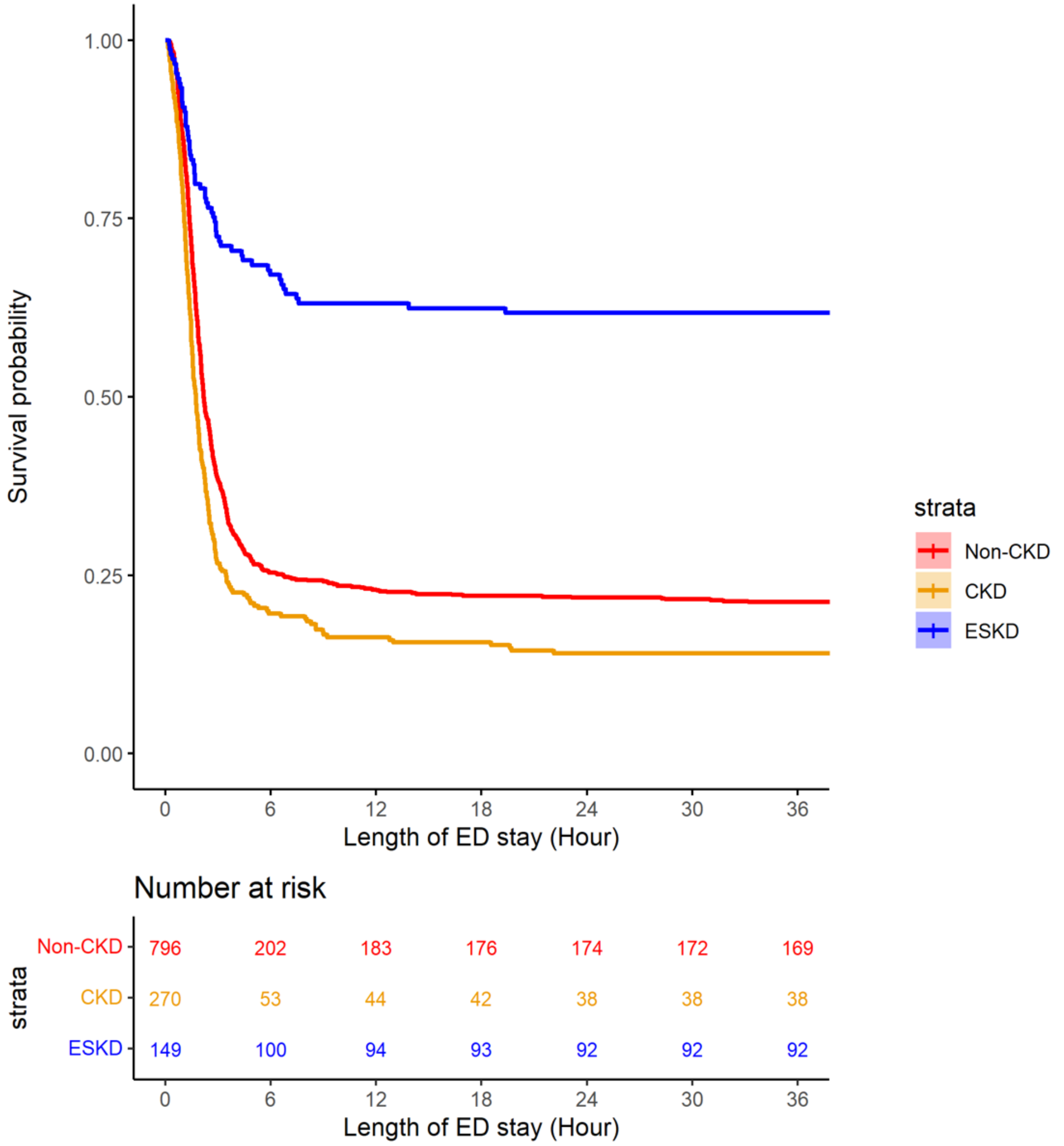

Figure S1. Kaplan–Meier survival curve used to evaluate the effect of end-stage kidney disease (ESKD) on the rate of attaining sustained return of spontaneous circulation (ROSC) in patients with out-of-hospital cardiac arrest (OHCA) for the evaluated time point, emergency department (ED) stay (survival until hospital admission). Patients without ESKD (red). Patients with ESKD (blue). Patients without CKD (orange). The x-axis shows the time in hours, and the y-axis shows the survival probability (ROSC).

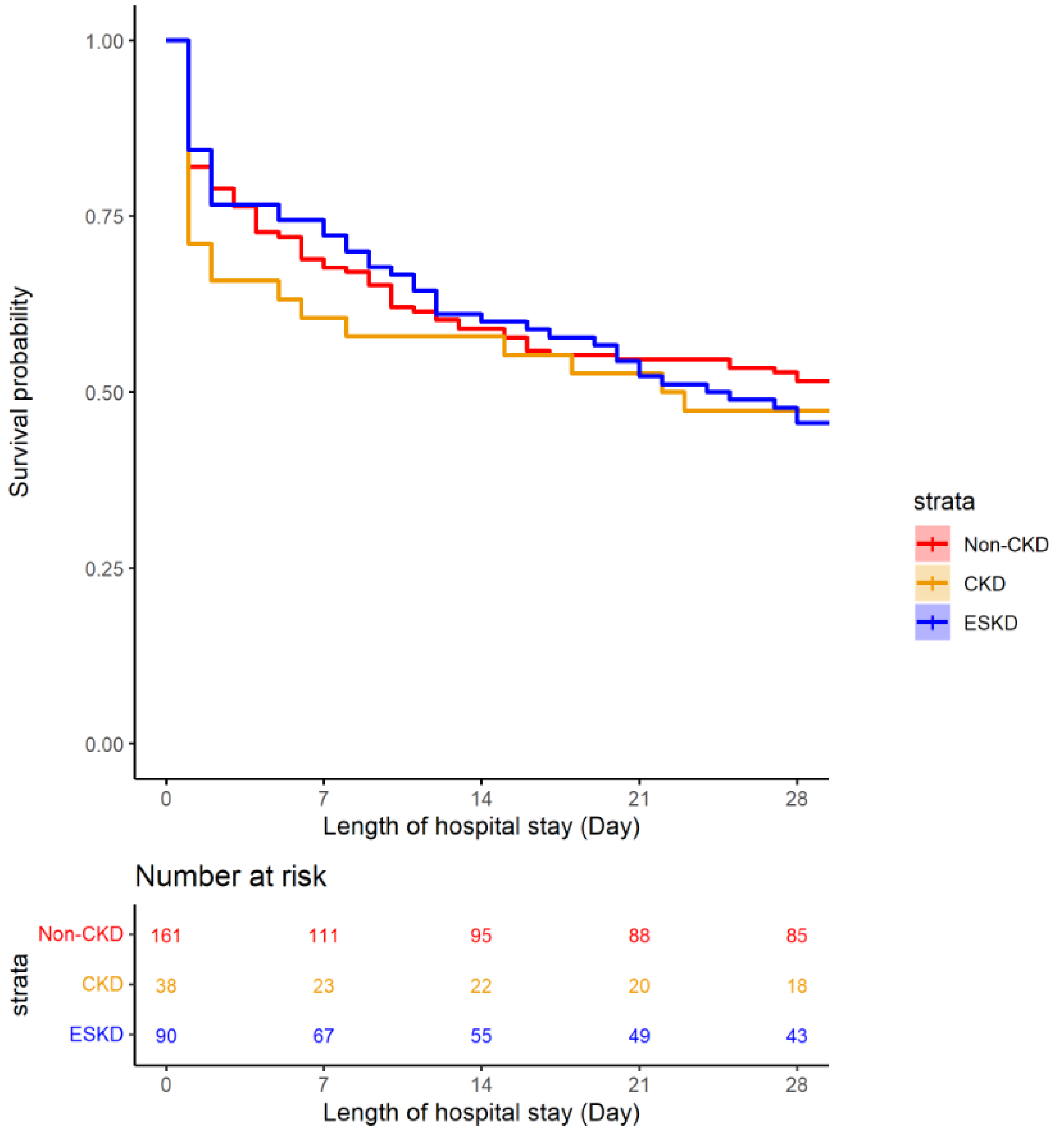

Figure S2. Kaplan–Meier survival curve used to evaluate the effect of end-stage kidney disease (ESKD) on the 28-day hospital survival in patients with sustained return of spontaneous circulation (ROSC). Patients without ESKD (red). Patients with ESKD (blue). Patients without CKD (orange). The x-axis depicts the number of days of hospital stay, and the y-axis shows the survival probability (ROSC).
